# Two-dimensional speckle tracking of the ascending aorta: a novel approach to evaluate arterial stiffness in pediatric patients with repaired conotruncal anomalies using echocardiography

**DOI:** 10.3389/fcvm.2025.1555568

**Published:** 2025-03-20

**Authors:** Anmol Goyal, Anitha Parthiban, David A. White, Daniel Forsha, Doaa Aly

**Affiliations:** ^1^ Ward Family Heart Center, Children’s Mercy Hospital, Kansas City, MO, United States; ^2^ Department of Pediatrics, University of Missouri-Kansas City School of Medicine, Kansas City, MO, United States; ^3^ Department of Pediatric Cardiology, Texas Children’s Hospital, Baylor College of Medicine Houston, Houston, TX, United States; ^4^ Benioff Children’s Hospital, University of California San Francisco, San Francisco, CA, United States

**Keywords:** speckle tracking echocardiography, arterial stiffness, aortic strain, conotruncal abnormalities, pediatrics

## Abstract

**Background:**

Children with repaired conotruncal abnormalities (CTA) are at risk of progressive aortic dilation and deteriorating aortic elasticity even after surgical correction. Strain imaging, using a 2-dimensional speckle tracking echocardiography (2D-STE), has been used for arterial stiffness assessment, but pediatric data are still lacking. We investigated the feasibility, reproducibility, and clinical value of 2D-STE derived ascending aorta (AAo) stiffness in children with repaired CTA.

**Methods:**

22 pediatric patients with repaired CTA were included along with 25 age- and sex- matched healthy controls (mean age 10.2 ± 4.5 years). Conventional 2D echocardiographic and 2D-STE assessment of AAo mechanics was performed. M-mode AAo strain, aortic distensibility and aortic stiffness index as well as 2D-STE derived AAo global circumferential strain (GCS) were calculated and compared between groups.

**Results:**

2D-STE was successfully performed and analyzed in the entire, prospectively enrolled cohort. Patients with repaired CTA had significantly lower 2D-STE derived AAo GCS compared to controls (9.4 ± 1.3 vs. 15.2 ± 1.7, *P*-value <0.001). AAo GCS in repaired CTA patients had strong negative correlation with age (*r* = −0.76, CI −0.9 to −0.51) and a moderate negative correlation with the absolute aortic valve annulus (*r* = −0.55), absolute aortic root (*r* = −0.67), absolute sino-tubular junction (*r* = −0.67) and absolute AAo dimension (*r* = −0.71). On multivariate analysis, absolute aortic root and ascending aorta dimension were significantly associated with AAo GCS independent of other variables. Intra-observer reproducibility was good to excellent for the CTA and entire cohort (ICC = 0.87 and 0.96 respectively), while inter-observer reproducibility was moderate for the CTA cohort 0.71 vs. 0.92 for the entire cohort.

**Conclusion:**

AAo GCS using 2D STE is highly feasible and reproducible as well as provides novel insights into the aortic deformation mechanics in pediatric patients with repaired CTA, thus holds promise in longitudinal assessment and risk stratification of aortopathy-associated congenital heart disease patients.

## Introduction

Conotruncal anomalies (CTA) account for 5%–10% of all congenital heart diseases, encompassing a diverse range of defects, such as tetralogy of Fallot (ToF), truncus arteriosus (TA), double outlet right ventricle (DORV), and transposition of the great arteries (TGA) (1). Aortopathy, a recognized comorbidity of CTA, stems from both histological and hemodynamic changes, including medial necrosis of the aortic wall and volume overload prior to surgical correction. These alterations can lead to significant perturbations in the mechanical characteristics of the aortic wall resulting in increased aortic stiffness and aortic wall shear stress, even after optimal surgical correction (2–4).

The spectrum of aortopathy in individuals with CTA often presents as enlargement of aortic root and ascending aorta. While less common, complications such as aortic aneurysm, dissection and valve dysfunction have been also reported in the adult population post- CTA repair (5–11). The heightened aortic stiffness and resultant decline in arterial elasticity can precipitate a left ventricular-afterload mismatch (i.e., ventriculo-arterial decoupling) (12) with subsequent deterioration in both the systolic and diastolic function of the left ventricle (LV) (13). Recognizing aortopathy as an independent predictor for subsequent cardiovascular events and mortality (14) as well as the aforementioned complications, it is crucial to implement early detection and precise characterization of progression of aortopathy in the clinical management of patients with CTA.

Echocardiography is the cornerstone imaging modality for clinical evaluation and ongoing outpatient monitoring of patients with CTA, hence, it is well suited for regular assessment of aortic stiffness in these patients. Speckle tracing echocardiography (STE) is an innovative echocardiographic method for quantification of aortic strain offering insights into the biomechanical behavior of the aortic wall (15). STE allows for segmental and global analysis of arterial strain with minimal dependence on angle of interrogation. It has been validated by histological, sonomicrometric and, more recently, MRI studies, and deemed a reliable representation of aortic wall stiffness in adults with aortic aneurysms (16, 17). Despite its proven effectiveness in adults, there is a notable gap in research regarding the use of STE for measuring aortic strain in pediatric patients with congenital heart disease, particularly those with CTA (18–23).

The aims of this study are: (1) To assess the feasibility and reproducibility of aortic strain by STE in prospectively enrolled children and adolescents with CTA. (2) To compare measures of aortic vascular mechanics by STE and other conventional echocardiography between patients with repaired CTA and age- and sex-matched healthy controls. (3) To examine the association between STE-derived aortic strain and demographic variables, physiologic parameters, left ventricular systolic function and aortic dimensions in study patients.

## Methods

### Design and participants

This is a prospective, single-center, case-control study. Participants aged 5–17 years old, presenting for routine follow up in the outpatient cardiology clinic were included. Cases consisted of patients with CTA, post complete surgical repair. Controls were age- and sex-matched participants referred to the outpatient cardiology clinic for variety of cardiac symptoms (chest pain, syncope, murmur, family history of heart disease, abnormal electrocardiogram, or suspected congenital heart disease) and had normal echocardiograms upon evaluation. Patients with known risk factors for abnormal ascending aorta properties such as those with aortic valve pathology, or connective tissue disorder, prior aortic root and or aortic valve interventions, obesity (BMI >30 kg/m^2^), stage II hypertension (>99th percentile for age) and history of dyslipidemia, diabetes or dysrhythmias were excluded. Medical history, physical examination, and electrocardiography were performed at each outpatient clinic visit. Participant demographics and clinical history were obtained from most recent cardiology clinic visits.

The study protocol conformed to the ethical guidelines of the 1975 Declaration of Helsinki and was approved by the Children's Mercy Kansas City Institutional Review Board. Informed consent and assent were obtained from participants and their legally authorized representatives prior to data collection.

### Conventional echocardiography

Transthoracic echocardiographic evaluation of the study cases and controls was performed using either Phillips EPIQ 7 or GE Vivd 95 ultrasound machines. Prior to the echocardiogram, right brachial blood pressure was measured using an electronic sphygmomanometer (Omron HBP-1100U; Omron Instruments, Kyoto, Japan) with the participant in a supine position.

Echocardiographic measurements including LV dimensions, LV ejection fraction, LV fractional shortening, LV mass index and aortic dimensions [aortic valve annulus, aortic root, sino-tubular junction, and ascending aorta (AAo)] were performed in accordance with the American Society of Echocardiography guidelines (24). *Z*-scores were derived from the Boston method based on body surface area (25, 26).

**Figure 1:**
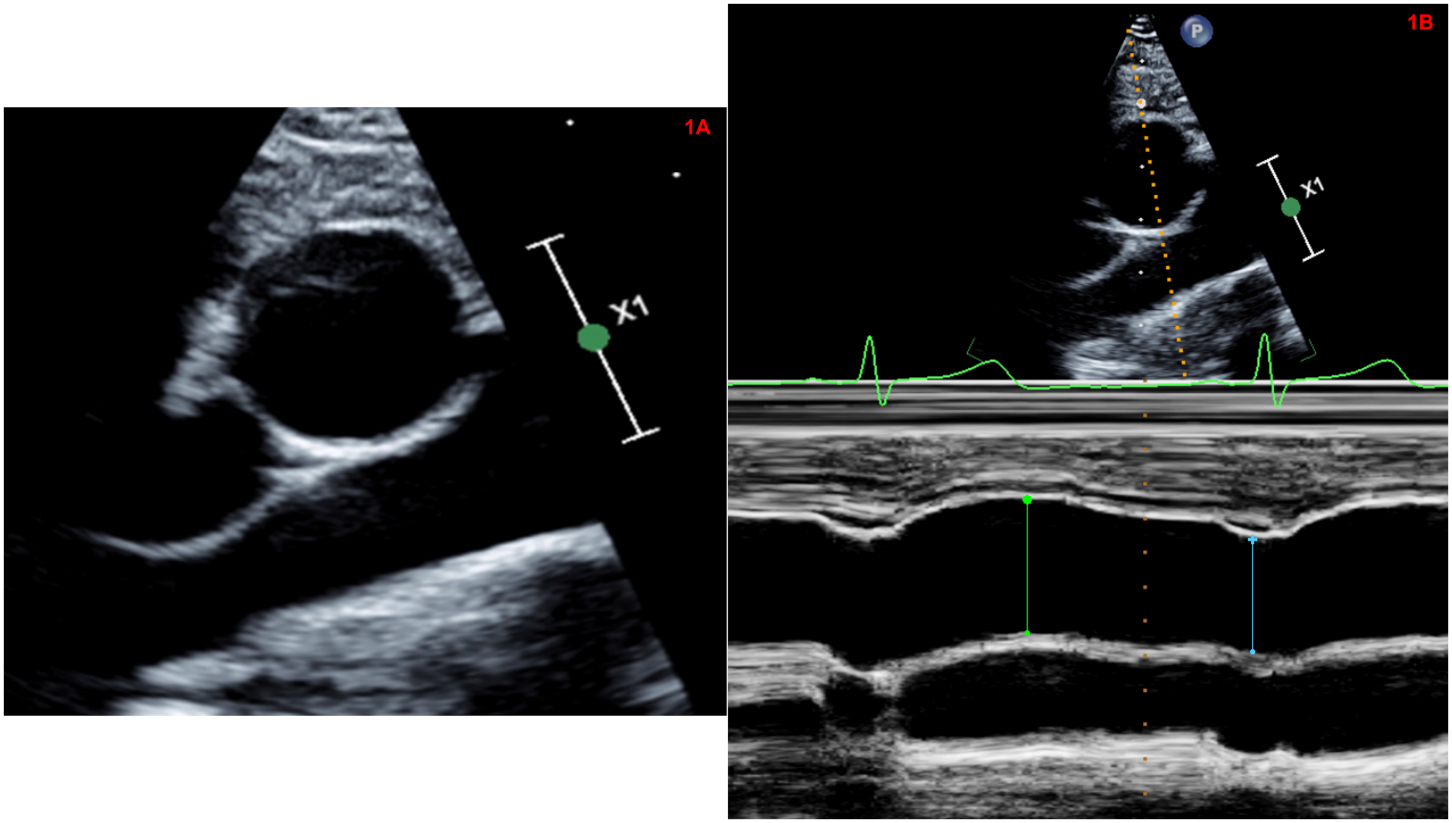
**(A)** Two-dimensional high right parasternal short axis view of the ascending aorta (at the level of the right pulmonary artery). **(B)** Two-dimensional guided M-mode of the ascending aorta from high right parasternal short axis view.

The following views were obtained at the end of each echocardiogram for assessment of aortic wall mechanics:

1. 2D parasternal short axis view of AAo (at the level of the right pulmonary artery) (Figure 1A).
2. 2D guided M-mode of AAo from parasternal short axis view (Figure 1B).
3. 2D short axis view of the aortic valve to assess aortic valve morphology.

The acquisition of these views was performed with breath holding to minimize translational motion of the vessel wall. Gain, compression and depth were optimized to define the vascular borders and ensure a frame rate of >30 frames/second. Loops of 3 cardiac cycles were stored as non-DICOM (native data) images. Image quality was assessed for each of the above views and graded based on the visualization of the inner border of the aortic wall on a scale of 1–3 by the primary investigator (AG) (1 = poor visualization; 2 = partial visualization; 3 = good visualization). The same observer (AG) who was blinded to the clinical status of the participants performed all measurements.

### Aortic mechanics by conventional M-mode echocardiography

Using 2D guided M-mode of the parasternal AAo short axis views, systolic aortic diameter (AoS) was measured at the point of maximal anterior motion of the AAo (systole), while diastolic aortic diameter (AoD) was measured at the Q wave on electrocardiogram (end diastole). Three different indices of aortic stiffness (aortic strain, aortic distensibility and aortic stiffness index) were calculated using previously published standard formulae (27, 28).

$$M\text{-}\mathrm{mode}\;\mathrm{Aortic}\;\mathrm{strain}\;=\;\frac{\mathrm{AoS}\text{-}\mathrm{AoD}}{\mathrm{AoD}}$$

$$M\text{-}\mathrm{mode}\;\mathrm{Aortic}\;\mathrm{distensibility}\;=\;\frac{2\frac{\left(\mathrm{AoS}\text{-}\mathrm{AoD}\right)}{\mathrm{AoD}}}{\mathrm{PP}}$$

$$M\text{-}\mathrm{mode}\;\mathrm{Aortic}\;\mathrm{stiffness}\;\mathrm{index}\;=\;\frac{\ln\left(\frac{\mathrm{SBP}}{\mathrm{DBP}}\right)}{\frac{\mathrm{AoS}\text{-}\mathrm{AoD}}{\mathrm{AoD}}}$$

AoS, aortic diameter in systole; AoD, aortic diameter in diastole; PP, pulse pressure; SBP, systolic blood pressure; DBP, diastolic blood pressure; ln SBP/DBP, natural logarithm of relative pressure.

### Aortic mechanics by 2D-STE

**Figure 2:**
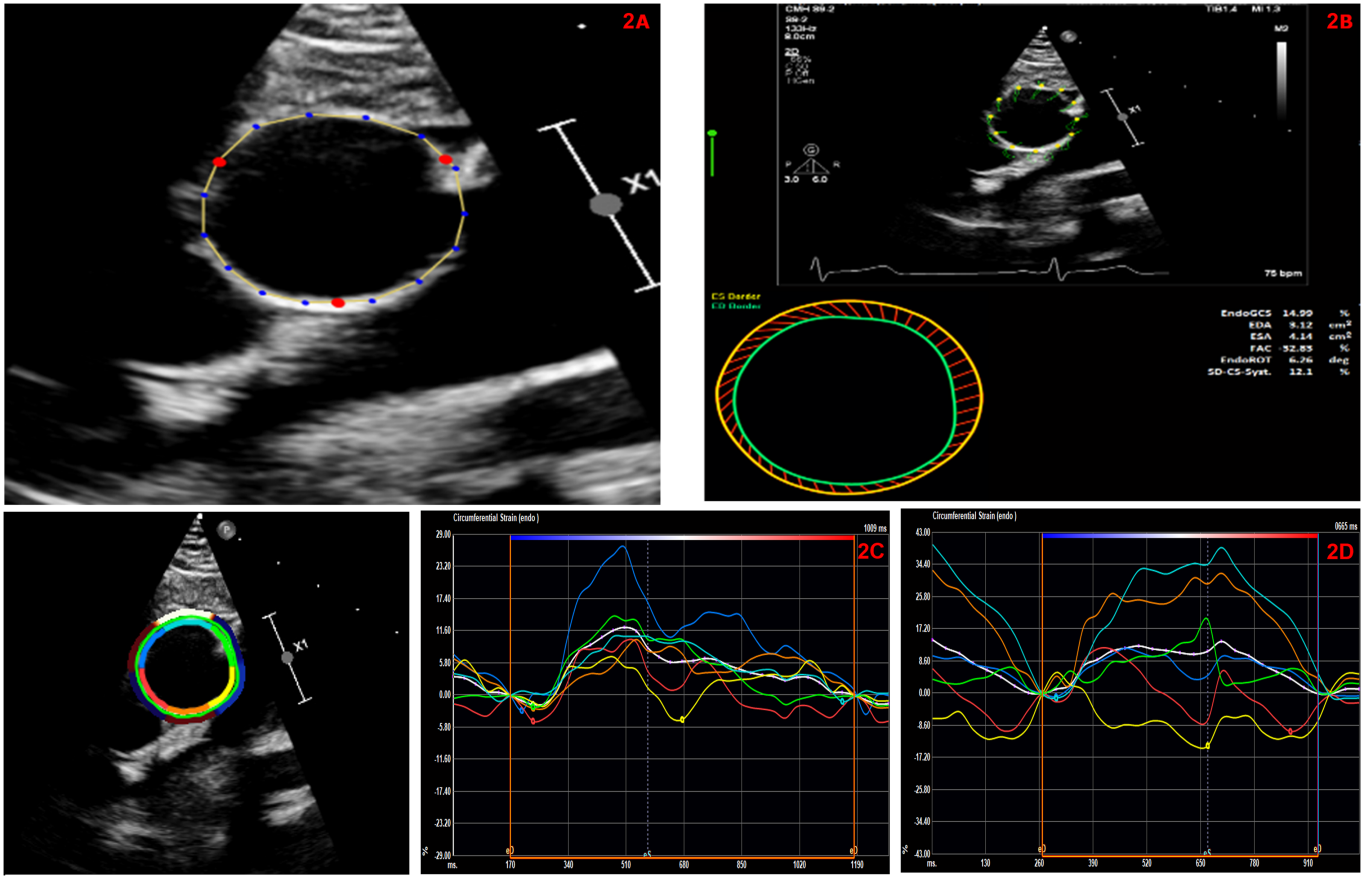
**(A)** Automated contour tracing to track the inner border of the ascending aortic wall using parasternal short axis view. **(B)** Automated global circumferential strain calculated by averaging the peak circumferential strains of the 6-segmented aortic model and expressed as an absolute value. **(C)** Representative AAo-GCS curves in a patient with repaired conotruncal abnormality. This patient had a AAo-GCS of 7.6%. **(D)** Representative AAo-GCS curves in an age and gender matched healthy control. This patient had a Aao-GCS of 12.7%.

2D short axis parasternal images of the AAo at the level of the right pulmonary artery (at the same sampling location of the M-mode echocardiographic measurements) were used for aortic strain measurements by STE. Post-processing was performed using commercially available software (Tomtec Cardiac Performance Analysis version 1.2 (CPA, Unterschliessheim, Germany). We used the LV global circumferential strain algorithm for assessment of aortic wall mechanics given the lack of a dedicated arterial wall strain algorithm. Using this vendor-independent software and advanced automated contour tracing (ACT), three specific landmarks were identified (10 o'clock, 2 o'clock and 6 o'clock position) to track the inner border of the aortic wall at the end systolic frame (Figure 2A). Region of interest was generated by ACT using a two-step knowledge-based algorithm.

Prior to processing, we visually confirmed that the ACT followed the inner border of the aortic wall throughout the cardiac cycle on a cine loop preview. If tracking was suboptimal, manual adjustments of the region-of-interest were attempted at a maximum of three times. The software performed speckle tracking analysis on a frame-by-frame basis and generated time-domain volume curves and circumferential strain profiles for six equally sized, color-coded segments (yellow, light blue, green, purple, blue, and red). Quantitative curves with different colors were used to represent the 2D-STE variables of different segments of the aortic wall. Peak AAo strain by STE, which typically appeared near the aortic valvular closure (systole), was identified and global circumferential strain (GCS) was calculated by averaging the peak circumferential strains of the 6-segment aortic model and expressed as an absolute value (Figure 2B).

### Feasibility and reproducibility AAo mechanics by 2D STE

Feasibility of AAo GCS was determined based on the total number of studies with successful STE tracking and analysis at less than or equal to three attempts of manual adjustments of ACT. With regards to reproducibility, retrospective offline analysis of AAo GCS was repeated by the same examiner (AG) after 4 weeks in 25% of randomly selected cohort for intra-observer reproducibility. For inter-observer reproducibility, AAo GCS analysis was performed by a second blinded examiner (DA) in the same 25% of randomly selected cohort and compared to the primary examiner (AG).

### Statistical analysis

Normal distribution was assessed using the Shapiro–Wilk test and checked visually from histograms. Characteristics of our cohort and controls were summarized as percentages, means ± standard deviation (SD) or as median, minimum, and maximum, as appropriate depending on the normalcy of the data. Categorical data were compared using chi-square test or Fischer-exact test for smaller sample groups (*n* < 5). The independent *T*-test and Wilcoxon rank sum test were used to compare normally and non-normally distributed continuous variables respectively to examine differences between our repaired CTA cohort and control group. The Pearson's correlation coefficient was calculated to determine the correlation between AAo GCS and physiological parameters as well as dimensional characteristics of the aortic vessel and left ventricle measured by transthoracic echocardiography. Multivariate analysis was performed to determine association between these variables and AAo GCS.

Intra- and inter-observer reproducibility of measurements was assessed by calculating the absolute agreement two-way random effects model intraclass correlation coefficient (ICC). ICC estimates and their 95% confident intervals (CI) are reported. Reproducibility was considered poor if ICC <0.50, moderate if 0.50–0.75, good if 0.75–0.90, and excellent if >0.90. (Portney et al.). All statistical analyses were performed utilizing SPSS 24 (IBM SPSS Statistics for Windows, Version 24.0. IBM Corp., Armonk, NY, USA, 2016) with *p*-value of ≤0.05 considered statistically significant.

## Results

A total of 22 participants with repaired CTA and 25 age- and sex -matched healthy controls were included. Demographic characteristics of the entire cohort are displayed in Table 1. Briefly, the mean age of the entire sample was 10.4 ± 4.1 years, the most common CTA was ToF (*n* = 16; 73%) and two-thirds of the cohort were males (*n* = 32; 64%). Among the case participants, 36% had positive pathologic genetic mutation with the most common being 22q11 deletions. Among the control participants with a normal echocardiogram, abnormal electrocardiogram (*n* = 7; 28%) was the most common indication for obtaining an echocardiogram, followed by chest pain (*n* = 6; 24%). There was no significant difference between the cases and controls with regards to body surface area, blood pressure and heart rate at the time of image acquisition.

**Table 1:** Participants demographics.

Participants characteristics	Cases	Control	*P*-value
Age at study (year)	10.2 ± 4.5	10.5 ± 3.8	0.81
Male sex (*n* and %)	14 (64%)	18 (72%)	0.55
BSA (m^2^)	1.2 ± 0.4	1.3 ± 0.4	0.35
SBP	108 ± 11.2	109 ± 10	0.77
DBP	61 ± 9.4	61 ± 8	0.94
Heart rate	73 ± 11	71 ± 12	0.52
Cardiac diagnosis (*n* and %)			
TOF	16 (73%)		
TGA	6 (27%)		
Age at repair (days)	167.2 ± 151.8		
Staged repair (*n* and %)	5 (23%)		

Results are displayed as mean ± SD unless otherwise described. Indications for referral in the control group included: Chest pain: 6 (24%); Murmur: 3 (12%); Syncope: 2 (8%); Family history of heart disease: 5 (20%); Abnormal electrocardiogram: 7 (28%); Suspected congenital heart disease: 2 (8%). BSA, body surface area; SBP, systolic blood pressure; DBP, diastolic blood pressure; TOF, tetralogy of Fallot; TGA, transposition of the GREAT arteries; TA, truncus arteriosus.

### General echocardiographic assessment

Conventional echocardiographic LV measurements, LV global longitudinal strain and aortic size measurements of both groups are detailed in Table 2. There were no significant between-group differences in ventricular volumes or function. Patients with repaired CTA had significantly larger aortic valve annulus, aortic root, sino-tubular junction, and AAo dimensions compared to controls, even when indexed to body surface area.

**Table 2:** General echocardiographic assessment parameters.

Echocardiographic parameters	Cases	Control	*P*-value
LVEDVi (ml/m^2^)	87.5 ± 22.8	81.7 ± 13.2	0.287
LVESVi (ml/m^2^)	35 ± 9.6	31.9 ± 7	0.209
Svi (ml/m^2^)	52 ± 16.4	49.8 ± 7.5	0.545
EF (%)	59.7 ± 5.2	61.1 ± 3.8	0.290
SF (%)	35.9 ± 4.7	36 ± 3.1	0.894
PWTd (cm)	0.7 ± 0.2	0.7 ± 0.2	0.155
IVSd (cm)	0.8 ± 0.2	0.7 ± 0.1	0.080
LVGLS %	−20.2 ± 1.1	−20.7 ± 1	0.097
Trileaflet aortic valve morphology (*n* and %)	22 (100%)	25 (100%)	
Aortic insufficiency (*n* and %)			
None	17 (77%)	25 (100%)	
Mild	5 (23%)		
Moderate	0 (0%)		
Severe	0 (0%)		
Aortic valve annulus (cm)	2.1 ± 0.4	1.8 ± 0.2	**0** **.** **005**
Aortic valve annulus *Z* score	2.4 ± 1.4	0.2 ± 0.9	**<0** **.** **001**
Aortic root (cm)	2.7 ± 0.5	2.2 ± 0.4	**0** **.** **001**
Aortic root *Z* score	2 ± 1	−0.5 ± 1	**<0** **.** **001**
Aortic STJ (cm)	2.2 ± 0.4	1.9 ± 0.3	**0** **.** **01**
Aortic STJ Z score	2 ± 1.1	0.05 ± 1	**<0** **.** **001**
Ascending aorta (cm)	2.4 ± 0.5	2.2 ± 0.3	**0** **.** **027**
Ascending aorta *Z* score	1.9 ± 1.4	0.2 ± 0.8	**<0** **.** **001**

*Results are displayed as mean* ± SD unless otherwise described. EF, ejection fraction; IVSd, interventricular septum thickness at end-diastole; LVEDVi, left ventricular end-diastolic volume indexed to BSA; LVESVi, left ventricular end-systolic volume indexed to BSA; LVGLS, left ventricular global longitudinal strain; PWTd, posterior wall thickness at end-diastole; SF, shortening fraction; SVi, stroke volume indexed to BSA.

Bold values represent statistically significant value of *p* < 0.05.

### Image visualization quality and feasibility and reproducibility of AAo GCS

None of the participants were excluded due to poor image quality. Image quality was graded as “good visualization quality” in 96% of participants (image grade 3, 45/47 participants) and “partial visualization quality” in 4% of participants (image grade 2, 2/47 participants). 2D-STE derived AAo GCS was successfully performed and analyzed for the entire cohort.

The intra-observer ICC of AAo GCS was 0.96 with 95% confidence intervals from 0.68 to 0.99, and the inter-observer ICC was 0.92 with 95% confidence intervals from 0.61 to 0.98, for the entire cohort (Table 3). When subdivided into repaired CTA cases and healthy controls, intra-observer ICC for AAo GCS was 0.87 with 95% confidence intervals from 0.55 to 0.98 for the repaired CTA group and 0.96 with 95% confidence intervals from 0.62 to 0.99 for the control group. Regarding inter-observer reproducibility, AAo GCS ICC was 0.71 with 95% confidence intervals from 0.56 to 0.96 for the repaired CTA group and 0.94 with 95% confidence intervals from 0.57 to 0.99 for the control group (Table 3).

**Table 3:** Intra-Class correlation coefficient: Two-way random effects model.

Ascending aorta GCS	Intra-observer	Inter-observer
Whole cohort	0.96 (0.68–0.99)	0.92 (0.61–0.98)
Cono-truncal group	0.87 (0.55–0.98)	0.71 (0.56–0.96)
Control group	0.96 (0.62–0.99)	0.94 (0.57–0.99)

### AAo mechanics by 2D-STE and M-mode

**Figure 3:**
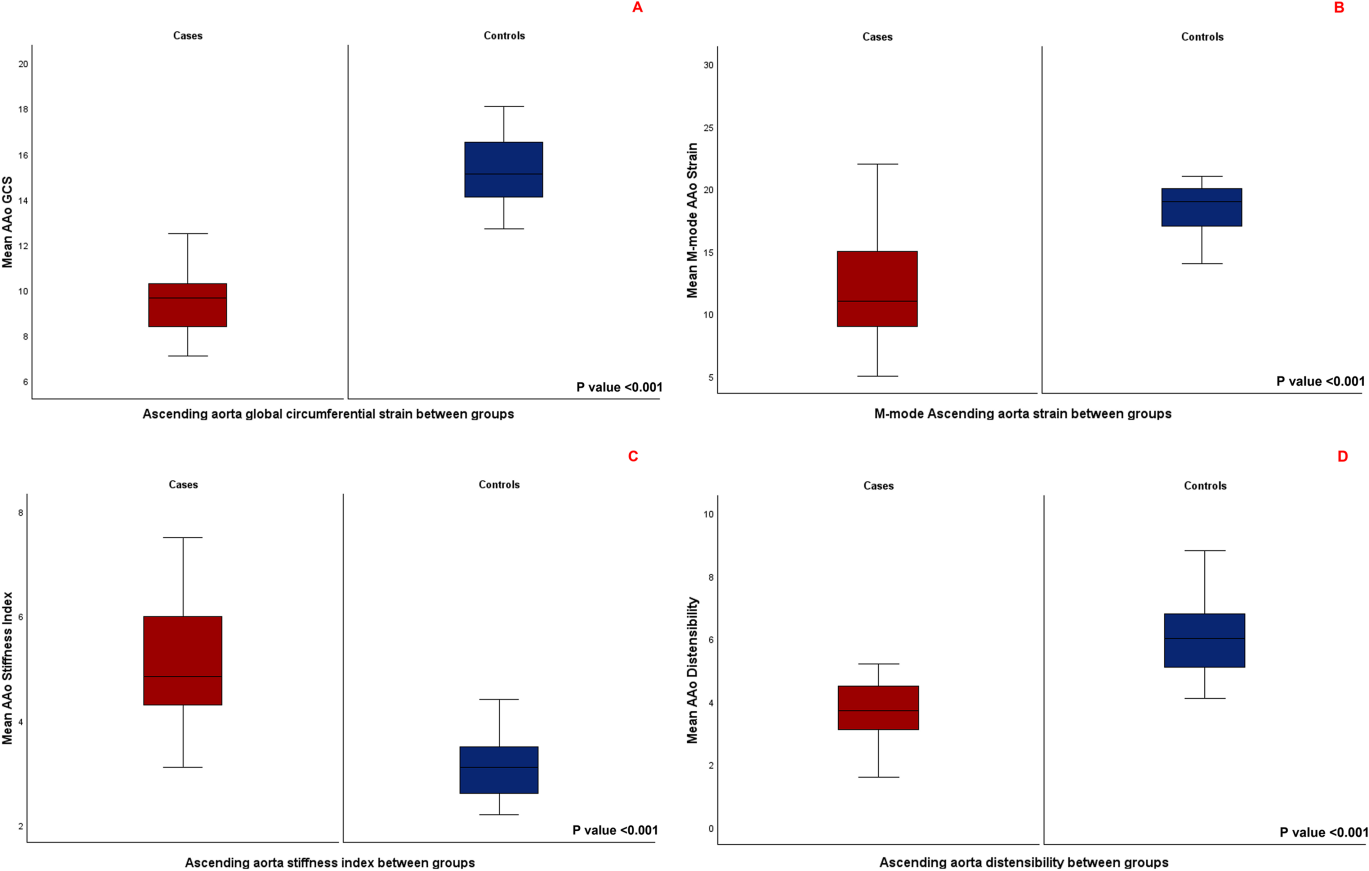
**(A)** Comparison of mean ascending aorta global circumferential strain between the two groups. **(B)** Comparison of mean M-mode derived ascending aorta strain between the two groups. **(C)** Comparison of mean ascending aorta stiffness index between the two groups. **(D)** Comparison of mean ascending aorta distensibility between the two groups.

AAo GCS assessed by 2D-STE was significantly lower in patients with repaired CTA compared to age- and sex matched controls (9.4 ± 1.3 vs. 15.2 ± 1.7, *P*-value <0.001), (Figure 3A). Aortic strain and aortic distensibility by M-mode were also significantly lower in patients with repaired CTA compared to matched controls (11.9 ± 4.5 vs. 19 ± 3.6, *P* < 0.001; 3.8 ± 1.1 vs. 6 ± 1.3, *P* < 0.001, respectively). Aortic stiffness index was significantly higher in patients with CTA compared to matched controls (5.2 ± 1.7 vs. 3.2 ± 0.6, *P*-value <0.001) (Figures 3B–D). AAo GCS by STE correlated strongly with the M-Mode measures of AAo strain in the entire cohort and moderately to strongly in the individual cases and matched controls subgroups (Table 4).

**Table 4:** Correlation of AAo GCS with demographic and echocardiographic variables.

Patient characteristics and echocardiographic parameters	All participants *r* (CI)	Cases *r* (CI)	Controls *r* (CI)
Age	−0.32 (−0.56 to −0.04)	−0.76 (−0.9 to −0.51)	−0.82 (−0.92 to −0.63)
BSA	−0.22 (−0.47–0.08)	−0.76 (−0.9 to −0.51)	−0.76 (−0.89 to −0.52)
SBP	−0.22 (−0.47–0.08)	−0.55 (−0.79 to −0.16)	−0.58 (−0.79 to −0.24)
Age at repair		−0.25 (−0.61–0.19)	
Aortic valve annulus (cm)	−0.58 (−0.75 to −0.35)	−0.55 (−0.79 to −0.17)	−0.64 (−0.83 to −0.33)
Aortic valve annulus *Z* score	−0.54 (−0.71 to −0.3)	0.09 (−0.34–0.49)	0.42 (0.03–0.7)
Aortic root (cm)	−0.67 (−0.8 to −0.48)	−0.67 (−0.85 to −0.35)	−0.69 (−0.85 to −0.41)
Aortic root *Z* score	−0.73 (−0.84 to −0.56)	−0.09 (−0.5–0.34)	−0.08 (−0.46–0.33)
Aortic STJ (cm)	−0.58 (−0.74 to −0.35)	−0.67 (−0.85 to −0.35)	−0.56 (−0.78 to −0.21)
Aortic STJ *Z* score	−0.6 (−0.76 to −0.38)	−0.27 (−0.62–0.17)	0.14 (−0.27–0.51)
Ascending aorta (cm)	−0.55 (−0.72 to −0.31)	−0.71 (−0.87 to −0.42)	−0.59 (−0.8 to −0.25)
Ascending aorta *Z* score	−0.56 (−0.73 to −0.33)	−0.27 (−0.62–0.17)	0.31 (−0.1–0.63)
LVEDVi	−0.22 (−0.47–0.08)	−0.09 (−0.49–0.35)	−0.29 (−0.62–0.12)
LVESVi	−0.24 (−0.49–0.06)	−0.08 (−0.49–0.35)	−0.24 (−0.58–0.17)
EF	0.15 (−0.15–0.42)	0.00 (−0.42–0.42)	0.03 (−0.37–0.42)
LVGLS	0.21 (−0.08–0.47)	−0.11 (−0.51–0.32)	0.05 (−0.35–0.44)
M-mode AAo strain	0.77 (0.62–0.87)	0.45 (0.03–0.73)	0.6 (0.26–0.8)
AAo distensibility	0.84 (0.74–0.91)	0.68 (0.37–0.86)	0.75 (0.5–0.88)
AAo stiffness index	−0.74 (−0.85 to −0.58)	−0.6 (−0.81 to −0.23)	−0.53 (−0.76 to −0.16)

EF, ejection fraction; LVEDVi, left ventricular end-diastolic volume indexed to BSA; LVESVi, left ventricular end-systolic volume indexed to BSA; LVGLS, left ventricular global longitudinal strain.

### Correlation between AAo GCS, physiologic parameters and aortic dimensions

**Figure 4:**
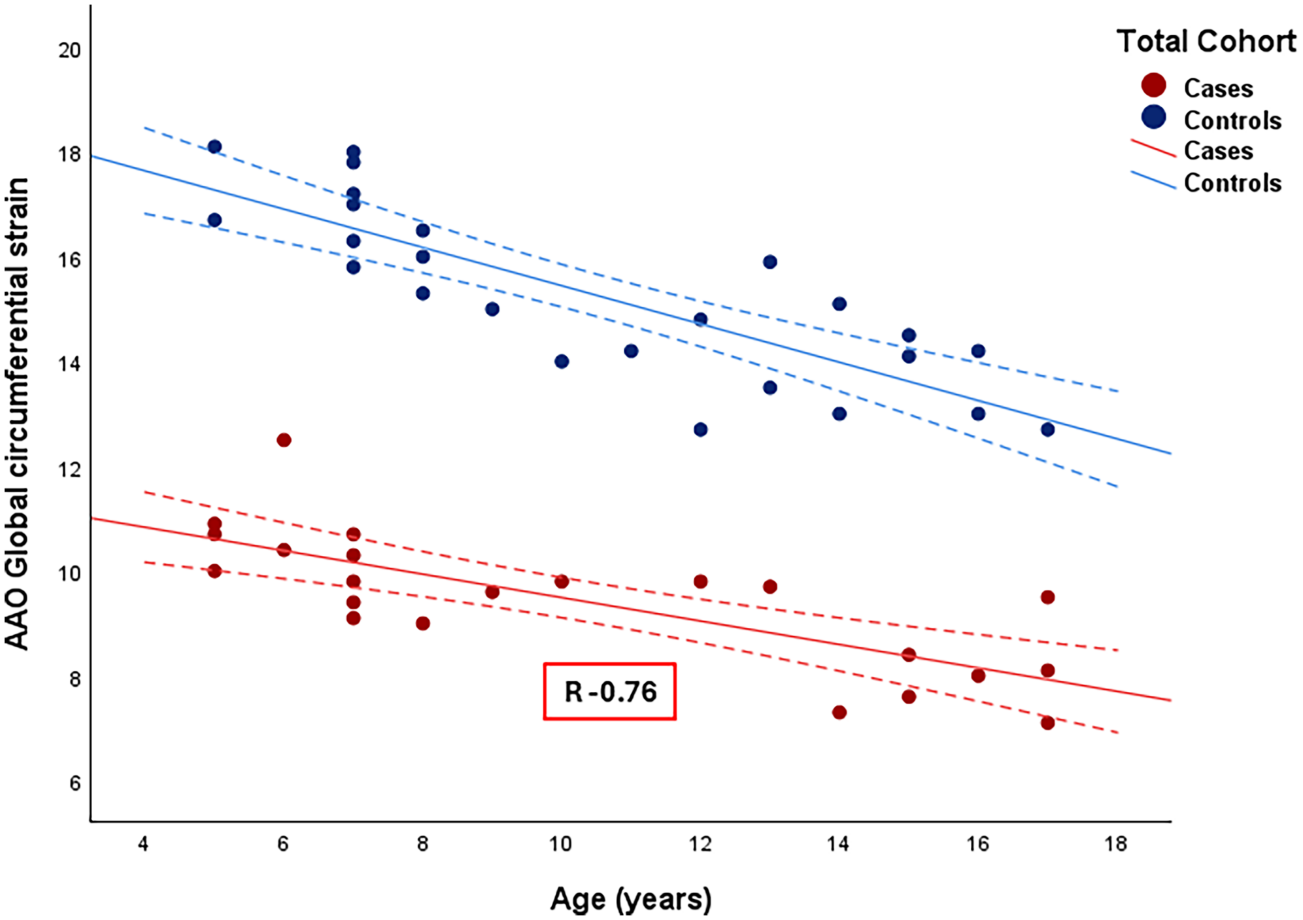
Correlation between ascending aorta global circumferential strain and age among the entire cohort. R: Correlation coefficient. Dotted line: 95% confidence interval.

Figure 4 displays the correlations between AAo GCS and age for the entire cohort (for cases, *r* = −0.76, CI −0.9 to −0.51). Among patients with repaired CTA, there was a strong negative correlation between AAo GCS and age, AAo GCS and BSA and moderate negative correlation with SBP (Table 4). The absolute aortic vessel dimensions namely, absolute aortic valve annulus, absolute aortic root dimension, absolute sino-tubular junction dimension, and absolute AAo dimension had a moderate negative correlation with AAo GCS (Table 4). No significant correlation was appreciated between AAo GCS and aortic dimensions when aortic *Z*-scores were used or between AAo GCS and measures of ventricular volumes and function in our study cohort (Table 4). On multivariate analysis, there was a significant correlation between absolute aortic root measurement and absolute ascending aorta measurements with GCS independent of other variables (Table 5).

**Table 5:** Multivariate analysis of Aao GCS within cases.

Patient characteristics and echocardiographic parameters	*R* ^2^	*P*-value
Age	0.97	0.12
BSA	0.70	0.1
SBP	0.78	0.8
HR	0.89	0.47
Age at repair (days)	0.91	0.36
Aortic valve annulus (cm)	0.98	0.07
Aortic valve annulus *Z* score	0.88	0.51
Aortic root (cm)	0.98	**0** **.** **04**
Aortic root *Z* score	0.60	0.98
Aortic STJ (cm)	0.97	0.11
Aortic STJ *Z* score	0.93	0.3
Ascending aorta (cm)	0.99	**0** **.** **015**
Ascending aorta *Z* score	0.77	0.82
EF	0.91	0.36
LVGLS	0.97	0.1

BSA, body surface area; SBP, systolic blood pressure; HR, heart rate; STJ, sino-tubular junction; EF, ejection fraction; LVGLS, left ventricular global longitudinal strain.

Bold values represent statistically significant value of *p* < 0.05.

## Discussion

To our knowledge, this is the first pediatric study to investigate the use of 2D-STE in assessing aortic vascular mechanics in children with repaired CTA. Our primary findings include:

a. Assessment of aortic stiffness by 2D-STE derived AAo GCS was highly feasible and reproducible among our study cohort.
b. 2D-STE derived AAo GCS was significantly lower in patients with repaired CTA compared to age- and sex-matched controls.
c. 2D-STE derived AAo GCS had strong negative correlation with age and body surface area, moderate negative correlation with blood pressure and absolute aortic measurements, and moderate to strong correlation with the M-Mode measures of aortic stiffness in patients with repaired CTA.

Patients with CTA are at risk of progressive aortopathy, with manifestations potentially emerging in infancy, even prior to the implementation of corrective surgical interventions (29). Aortopathy encompasses a spectrum of degenerative and fibrotic alterations within the medial layer of aortic wall, culminating in increased stiffness, dilation and possible aneurysmal transformation of the AAo. While CTA- associated aortopathy was traditionally regarded as a benign morphological anomaly, evolving literature implicates potential pathology with risk of significant clinical consequences.

Increased aortic wall stiffness has been correlated with ventriculo-arterial uncoupling and afterload mismatch leading to reduced left ventricular function in both children and adults with repaired TOF (12, 13). This is thought to be related to secondary flow patterns consisting of complex supraphysiological vortical and helical flow structures with increased aortic stiffness, that increase energy dissipation and overall myocardial workload (3, 4).

The revelation that aortopathy may significantly impact the clinical trajectory in patients with CTA is congruent with existing literature that positions aortic stiffness as a critical prognostic factor across various cardiovascular pathologies, including coronary artery disease, congestive heart failure, and atrial fibrillation (30, 31). Consequently, the early detection and precise delineation of aortopathy's progression in CTA patients are imperative. Such proactive measures are essential to elucidate the long-term prognostic implications more accurately and to refine the therapeutic strategies, thereby enhancing the efficacy of both medical and surgical interventions for these individuals.

### Feasibility and reproducibility of AAo GCS in CTA patients

Aortic 2D-STE is a novel non-invasive echocardiographic tool for quantification of aortic wall mechanics. By tracking aortic echo-dense speckles, aortic 2D-STE provides an angle independent calculation of aortic wall motion and deformation. It permits segmental and global assessment of vascular circumference regardless of arterial wall thickness and has been validated by histological and sonomicrometric studies of circumferential vascular mechanics (17). Prior research has demonstrated feasibility and accuracy of 2D-STE derived aortic circumferential strain for quantification of vascular stiffness in adults, but data about its applicability in the pediatric CHD population is still lacking (18, 20–23, 32). Our study demonstrated excellent feasibility of aortic strain by STE in a prospectively enrolled pediatric cohort with CTA. This is notable given the inherent physiologic differences between adults and children with respect to heart rate, blood pressure, as well as differences in imaging technique (imaging target depth, and angle of insonation).

Furthermore, our data suggested excellent inter- and intra-observer reproducibility of AAo GCS in children and adolescents with normal cardiovascular anatomy and good inter- and intra-observer reproducibility among those with repaired CTA. The lack of significant inter- and intra-observer variability of our AAO GCS measurements was likely enhanced by the semi-automated STE tracking and analysis built- in algorithms, as well as our standardized image acquisition and strain analysis protocols. The slightly lower reproducibility observed among the repaired CTA group compared to normal could be secondary to the limited echocardiographic assessment of the proximal aorta in this population of patients. This can be related to the anterior and superior displacement of aortic root and AAo, aortic dilation, varying relationship with the pulmonary arteries and the limited parasternal acoustic windows following post-surgical interventions and absent thymic tissue (with 22q11 deletion) in patients with CTA.

### Aortic mechanics in patients with repaired CTA

Patients with CTA had significantly lower 2D-STE AAo GCS values compared to healthy controls suggesting impaired mechanics with a higher degree of stiffness. Concordantly, and in agreement with previously published studies, patients had worse conventional M-mode markers of aortic stiffness compared to their age- and sex-matched healthy controls (12, 33, 34). As previously described, progressive aortic stiffness in patients with repaired CTA is likely a consequence of complex histological (medionecrosis, fibrosis and elastic fragmentation) and pathophysiological implications of abnormal load bearing, volume, shear and metabolic stresses and additional effects of abnormal aortic geometry following surgical repair (2, 35–41).

While 2D STE-derived aortic strain seems to have reached the same conclusion as by M-mode analysis, STE can be regarded as a potentially superior modality given its ability to provide more detailed and broader analysis of aortic mechanics than conventional M-Mode analysis. Unlike M-mode (where only a particular segment of the AAo is being evaluated), STE AAo GCS can be measured in all segments along the aortic circumference. This may provide a more precise assessment of arterial stiffness than M-Mode echocardiography given the heterogeneity of AAo arterial wall composition, particularly in patients with CTA (42, 43).

### Association between AAo GCS and demographic and physiologic variables

Our data demonstrated negative correlation between AAo GCS and age which is congruent with several prior studies demonstrating progressive aortic stiffening with age despite surgical repair (12, 29, 44). The aortic elastic properties depend largely on the presence of elastic fibers in the vessel wall, which have a maximal concentration in the perinatal period followed by a rapid decline during childhood and undergo further changes secondary to cyclic mechanical stress resulting in a stiffer aorta (45). In patients with CTA, this process might be further compounded by the intrinsic histological abnormalities and volume overload resulting in much stiffer AAo compared to their age-matched control as seen in our study. Also, and similar to the review by Savant et al. (46) we demonstrated significant negative correlation between AAo GCS and body surface area and systolic blood pressure. It is worth mentioning that within the present study, we sought to limit the effect hypertension, obesity and significant aortic valve insufficiency may have on the calculation on aortic wall stiffness by limiting our patient cohort to only those with normal blood pressure and body mass index and mild or less aortic valve insufficiency.

Our results also suggested significant correlation between the absolute size of the aortic root, aortic sino-tubular junction and AAo and degree of aortic wall stiffness in patients with repaired CTA. This is similar to the studies by Chong et al. (33) and Takei et al. (47), suggesting a complex interplay between increased aortic stiffness and progressive aortic root dilation in this particular subset of congenital heart disease. Although, normative data for pediatric 2D-STE derived AAo GCS are not currently available, given its good reproducibility and high correlatability, we propose that individual data can serve as their own baseline for longitudinal assessment of aortic stiffness in patients with CTA.

### Association between AAo GCS and left ventricular indices in repaired CTA

Increased aortic wall stiffness was shown to be associated with impaired ventricular-arterial coupling resulting in reduced left ventricular systolic and diastolic function in adults with repaired TOF (12, 13). In the present study, there was no significant correlation between the LV indices and increased aortic stiffness, although this is in the setting of normal ventricular function and volumes at baseline. This might be secondary to the relatively younger age of our patients compared to the previously published studies (48–51), where the deleterious effects of aortic stiffness on ventricular function might be lagging, as well as the small sample size which was not particularly powered to examine this specific association. In a prior study by Stone et al., a pediatric group of patients with repaired truncus arteriosus underwent detailed assessment of central aortic stiffness and LV mechanics by cardiac MRI. The authors demonstrated evidence of increased aortic stiffness, as well as impaired LV systolic and diastolic function, yet with no significant correlation between examined LV functional indices and aortic mechanics (52). Neither study was powered to test the correlative or causative relationships between adverse aortic remodeling and LV dysfunction. Dedicated longitudinal studies are thus warranted within the CTA patient population to further evaluate chronologic aortic biomechanical properties and to concurrently evaluate functional characteristics and volumetric parameters of LV function as they pertain to aortic biomechanics. With the recent advances and wide-spread adoption of STE imaging, STE provides a feasible, readily available tool for ongoing combined assessment of myocardial and aortic mechanics of the growing population of repaired CTA patients.

### Limitations

The present study is limited by its observational nature, therefore causal data about clinical implications of impaired aortic strain by STE were not recorded. Validation of aortic strain by STE against the gold standard measures of aortic stiffness (pulse wave velocity or cardiac MRI) was not performed in this study therefore we cannot confirm superiority of one measure of aortic stiffness over the other (STE vs. M-mode). This study was rather designed to establish feasibility and reproducibility of the novel aortic strain by STE in a presumably challenging population of patients. Finally, normal references for pediatric STE derived AAo strain are currently unavailable, therefore the degree of deviation from normal could not be ascertained. This should be pursued in further studies with larger populations from across different age groups, gender and cardiovascular anatomy to assess known detrimental effects of progressive aortic stiffness on cardiovascular physiology.

## Conclusion

2D-STE evaluation of aortic wall mechanics by AAo GCS is feasible in this pediatric patient population, with high degree of reproducibility. Even though longitudinal data correlating deranged arterial health in childhood with cardiovascular morbidity and mortality are lacking, one could consider using this novel noninvasive testing modality for longitudinal assessment and its potential role for risk stratification of aortopathy-associated congenital heart disease patients in the future.

## Data Availability

The original contributions presented in the study are included in the article/Supplementary Material, further inquiries can be directed to the corresponding author.
